# Quantum psychology approach on enjoyment as mediator in relationship between L2 flow and engagement

**DOI:** 10.3389/fpsyg.2025.1593973

**Published:** 2025-07-21

**Authors:** Ferdi Çelik

**Affiliations:** Department of English Language Education, Ondokuz Mayıs University, Samsun, Türkiye

**Keywords:** quantum psychology, SLA, flow and engagement, EFL, foreign language enjoyment, non-linear dynamics, mediation

## Abstract

**Introduction:**

Quantum psychology offers scholars a novel lens, to be preferred when Chaos/Complexity falls short of understanding the missing puzzle pieces in the complex interplay between cognitive and affective factors in second language acquisition (SLA). Adopting a quantum psychology approach, this study conceptualizes learner psychology as probabilistic and non-linear, proposing that flow, enjoyment, and engagement are dynamically entangled, akin to quantum principles of superposition and entanglement.

**Methods:**

To test this, the study investigates the mediating role of enjoyment in the relationship between psychological flow and academic engagement among 162 high school students in artificial intelligence (AI)-assisted English as a Foreign Language (EFL) speaking and writing classes. Employing a quantitative, cross-sectional design, data were collected using a validated Psychological Flow Scale, Academic Engagement Scale, and Foreign Language Enjoyment Scale.

**Results:**

Path analysis has revealed that psychological flow does not directly influence academic engagement; however, flow significantly predicts enjoyment, which in turn positively affects engagement. The indirect effect of flow on engagement through enjoyment was significant and indicated full mediation.

**Discussion:**

These findings challenge linear models of flow and engagement in SLA. The results, interpreted through a quantum perspective, emphasize the importance of fostering enjoyable learning experiences to enhance engagement, particularly in technology-enhanced contexts like AI-assisted classrooms. Pedagogical implications include designing interactive, enjoyable, and optimally challenging tasks to promote flow and enjoyment to sustain learner engagement. Future studies should investigate more mediators and use longitudinal designs to clarify these evolving relationships.

## 1 Introduction

In second language acquisition (SLA), learner psychology investigates the cognitive, emotional, and social factors that guide individuals in learning a second language (L2). Gaining insight into these psychological facets is pivotal for devising effective teaching strategies. Recent scholarship has highlighted psychological flow, enjoyment, and engagement as key constructs (Dewaele and MacIntyre, 2024; Hiver et al., 2024; Li et al., 2021). Engagement, situated at the intersection of emotion and cognition, is recognized as a strong predictor of language learning success (Zeng, 2021). Psychological flow, often portrayed as a stable and linear phenomenon directly linked to engagement (Jacobs and Morgan, 2022), has recently been questioned by emerging evidence (Feng and Hong, 2022). Meanwhile, enjoyment has gained prominence as a critical factor affecting learners’ motivation, resilience, and overall outcomes in language education (Hosseini et al., 2022). Research grounded in positive psychology shows that positive emotions, including enjoyment, lead to higher engagement and motivation (Hiver et al., 2024; Li et al., 2024; Zhang et al., 2024). Enjoyment promotes cognitive flexibility, boosts task persistence, and reduces anxiety (Guo, 2021). Yet, scholarly findings indicate flow does not always directly translate into engagement (Tsang and Dewaele, 2023), raising the question: Could enjoyment be the missing link that explains how flow fosters greater engagement in the L2 classroom? Moreover, the interdependence between enjoyment and engagement worth further exploration as two variables co-evolve dynamically rather than exist in isolation (Hosseini et al., 2022; Yang et al., 2023). Thus, given the limitations of traditional linear models and the deterministic chaos approach in fully capturing the complexities of learner psychology, an alternative framework is needed and it warrants a review of flow, enjoyment, engagement and an alternative theoretical lens.

### 1.1 Psychological flow

Psychological flow refers to a state of deep focus during an activity (Csikszentmihalyi, 1975; Csikszentmihalyi, 1990). Over two decades of experience-sampling research with more than 8,000 real-time self-reports collected from adolescents and adults in North America, Europe, and Asia enabled Csikszentmihalyi (1990) to distil *eight phenomenological markers* of optimal experience: (1) a perceived balance between skills and challenges, (2) clear, proximal goals, (3) unambiguous and immediate feedback, (4) intense yet effortless concentration, (5) a sense of control without conscious striving, (6) loss of self-consciousness, (7) altered time perception, and (8) an autotelic (intrinsically rewarding) payoff. Plotted together, these markers form the celebrated “flow channel” in which boredom (low challenge) and anxiety (excessive challenge) frame the sweet spot where engagement flourishes. Subsequent classroom work (Aubrey, 2017; Bodea and Trofimovich, 2023) consistently shows that when challenge–skill balance and clear feedback are engineered into language tasks, learners report the full phenomenological profile identified by Csikszentmihalyi. Our study builds directly on that lineage but probes a missing link: does the positive affect inherent in flow, operationalized here as foreign-language enjoyment, carry the motivational load that ultimately sustains academic engagement?

Researchers have recently studied its importance in language learning and concluded that experiencing “flow” can enhance learner engagement, motivation, and overall academic performance (Dewaele and MacIntyre, 2024; Jacobs and Morgan, 2022; Jahedizadeh et al., 2021). Engagement is also considered as a construct that has deep connections with flow (Mazid et al., 2024; Smith et al., 2023). Recruiting 1,044 language learners globally, Dewaele and MacIntyre (2024) has concluded that enjoyment, more than anxiety, significantly predicts the emergence of flow in language classrooms, and that flow becomes more frequent and socially shared as learners advance and feel more integrated. Wang and Huang (2022) investigate flow through enjoyment, boredom, and anxiety, as both the positive and negative dimensions of the learning experience intersects with flow. In their study Liu and Song (2020) emphasize that objectives need to be clear, and a balanced ratio of skills to challenges should be provided so as to achieve flow in online digital learning contexts. Meanwhile, Chen et al. (2021) discovered that genuine enthusiasm for language study, particularly harmonious passion, can heighten the sense of flow and encourage more active participation. Although these studies offer valuable insights, still, the precise ways in which flow, enjoyment, and engagement intersect remain an open question in need of further exploration.

### 1.2 Engagement

Engagement refers to individuals’ level of involvement during the learning process (Ghelichli et al., 2023). For students, academic engagement means how much effort they put into studying, attending classes, participating in discussions, completing assignments, and maintaining interest in their education, acting as a critical determinant in second-language (L2) acquisition (Hiver et al., 2024). Engagement is often considered a multifaceted construct. It reflects how various forms of engagement, including behavioral, emotional, and cognitive, contribute to second language acquisition (SLA). This is supported by research linking these dimensions to students’ L2 performance both inside the classroom (Alzahrani, 2025) and beyond it (Li, 2024).

Both what teachers do and what learners feel is important for engagement. Regarding task design, when activities are well-structured and matched to learners’ level of challenge, students display higher behavioral and cognitive engagement (Zare and Derakhshan, 2024). Multimodal tasks, for example pairing authentic audio with visual or interactive support, keep attention focused and deepen processing of the target language (García-Pinar, 2024). Turning to learner-trait factors, students who report stronger emotional engagement also report greater motivation, which in turn predicts superior L2 gains (Özhan and Kocadere, 2019). Likewise, higher emotional intelligence is associated with greater persistence and achievement in language study (Aljasir, 2024).

### 1.3 Enjoyment

Enjoyment in English language learning is a critical factor influencing various psychological constructs and L2 acquisition. Research indicates that both peer support and regulatory emotional self-efficacy serve as positive predictors of enjoyment, with self-efficacy functioning as an intermediary factor (Pan and Yuan, 2023). Enjoyment is also linked to learners’ ideal L2 selves, with private-internalized enjoyment playing a key role in shaping motivation and self-perceived proficiency (Kim, 2023). Based on our experiences and the studies in the literature, it can be stated that while motivation is more of a factor that the teacher might not easily shape in short-term interventions, enjoyment is rather more uncomplicated to be created by the teacher by adding pedagogical elements that are enjoyable for the learners or building a rapport with students (Kim, 2023; Thumvichit, 2022).

As such enjoyment in language learning is shaped not only by individual learner traits but also by external support systems, especially teacher involvement and instructional design. Teacher support plays a crucial role in enhancing enjoyment, as it helps foster students’ grit and long-term commitment to language acquisition (Hejazi and Sadoughi, 2022). However, this support must be reciprocal: to cultivate positive classroom environments, teachers themselves need institutional strategies and policies that promote their own wellbeing and job satisfaction (Demir et al., 2025). Beyond interpersonal factors, technological tools also contribute meaningfully. For instance, AI-assisted platforms have been shown to enhance learner enjoyment and improve academic outcomes (An et al., 2021). Real-time assessment tools such as the “enjoymeter” offer valuable insights into how enjoyment fluctuates during different learning phases (Wang, 2022). Collaborative learning strategies, particularly in online settings, can also heighten enjoyment by fostering peer support and a sense of shared purpose (Zheng and Zhou, 2022). Moreover, enjoyment helps mitigate language learning anxiety, especially in socially supportive classroom contexts (Fang and Tang, 2021). Finally, motivation has been identified as a critical mediator in this process, with higher levels of enjoyment strongly linked to increased motivation and improved English language achievement (Wang et al., 2023).

### 1.4 Chaos/complexity theory versus quantum theory

While the chaos/complexity theory of SLA (Larsen-Freeman, 2023) has long offered a unique lens to investigate the deterministic systems of learner psychology by proposing that learning has “sensitive dependence on initial conditions” (p. 408), it falls short in explaining probabilistic nature of SLA. For instance, while it may explain the non-linear, dynamic, and often chaotic nature of SLA (Larsen-Freeman, 2022), focusing on how small changes in pedagogy can lead to vastly different outcomes makes L2 instructions’ long-term predictability difficult (Borrero and Mariscal, 2021; Karaca and Baleanu, 2022; Lenzing et al., 2022). Understanding why flow does not always result in engagement, or why engagement does not consistently lead to improved L2 acquisition, guides scholars to investigate the individual variables that may influence these outcomes. In L2 contexts, this turns into a *deadlock*, where even a student with a cold, consistently sneezing, can affect the final outcome, affecting the learning experience. In other words, chaos/complexity theory with deterministic but unpredictable nature poses serious limitations in long-term understanding and the reason why in some L2 contexts, engagement and flow are not directly correlated in short, single-task studies (Goh and Yang, 2021; Zuniga, 2023), whereas semester-long classroom studies likewise reveal misalignment (Cho, 2019; Wang and Huang, 2022).

On the other hand, with its inherently probabilistic nature, quantum theory may equip SLA scholars with a tool necessary to understand these “complex” mechanisms. Using principles from quantum mechanics to human cognition, quantum psychology (Kyriazos and Poga, 2024) offers such a perspective by metaphorically suggesting that learner psychology exists in a *superposition* of cognitive states, where engagement is not a direct outcome of flow but rather an emergent property influenced by other factors (Pothos and Busemeyer, 2013; Feng and Hong, 2022). Some researchers propose that mental states may be likened to quantum principles, such as superposition and entanglement, which allows for a more dynamic and probabilistic understanding of cognitive processes (Busemeyer and Wang, 2015). By integrating quantum cognition models, SLA psychology can better explain cognitive biases, ambiguous mental states, and the interaction between the conscious and unconscious mind. Furthermore, quantum-based approaches offer insights into emotional regulation and mental health interventions, highlighting the role of consciousness in shaping reality and behavior (Shylesh, 2024). Therefore, the adoption of quantum psychology (Kyriazos and Poga, 2024) can add to our understanding of the mind and foster innovative research practices. Lastly, Csikszentmihalyi’s eighth marker autotelic reward dovetails with Fredrickson’s (2001) broaden-and-build proposition that positive emotions widen attentional scope and fuel subsequent action. In SLA settings, this reward is typically labeled foreign-language enjoyment (Dewaele and MacIntyre, 2014). We therefore conceptualize enjoyment as the affective core of flow and predict that it is this affect, rather than flow *per se*, that energizes sustained academic engagement.

### 1.5 Relevance of quantum psychology to SLA

Quantum cognition models propose that human mental states operate in ways analogous to quantum systems, where decisions and perceptions may exist in a superposition of possibilities until a cognitive “observation” (e.g., a choice or emotion) causes one outcome to emerge (Pothos and Busemeyer, 2013). Entanglement, in this context, refers to the idea that certain cognitive or emotional variables such as flow and enjoyment are not independent but co-vary in a tightly linked, non-separable way, meaning that a change in one instantly influences the other regardless of linear order (Busemeyer and Wang, 2015).

Quantum cognition treats mental states as probability amplitudes that “collapse” under observation (Pothos and Busemeyer, 2013). In language learning this implies (i) superposition—learners can simultaneously *intend* to engage and *hesitate*; (ii) entanglement—affect (e.g., enjoyment) and cognition (e.g., flow) are non-separable. Classic complexity theory models deterministic but unpredictable trajectories, whereas a quantum lens models *probabilistic* transitions. Our study operationalizes this by testing an indirect-only pathway in which flow’s effect on engagement *collapses* through enjoyment. Demonstrating a full mediation would therefore constitute preliminary empirical support for a quantum-psychology account.

### 1.6 The gap and the current study

**FIGURE 1:**
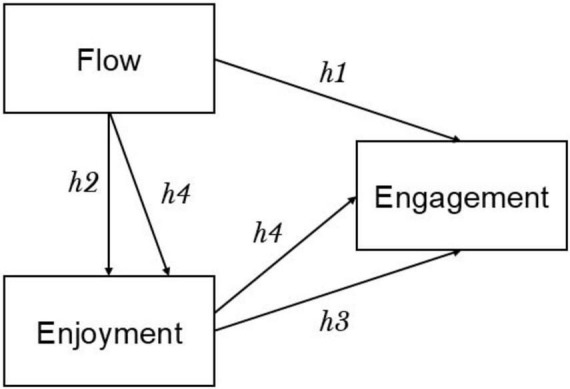
Hypothesized model.

First, our critical review of literature has shown that while chaos/complexity theory provides valuable insights into the non-linear dynamics of SLA, it is inherently deterministic, meaning that outcomes are theoretically predictable given complete knowledge of initial conditions (Larsen-Freeman, 2022). However, in practice, the complexity of SLA systems makes long-term prediction impossible due to their claim on sensitivity to initial conditions. This limitation is particularly problematic in understanding why flow does not always lead to engagement or why engagement does not always result in improved L2 acquisition (Tsang and Dewaele, 2023). Quantum theory, with its probabilistic nature, can offer a different perspective. It suggests that learner psychology exists in a superposition of states, where outcomes are not determined until observed, which allows for a more flexible understanding of the interplay between cognitive and affective factors. This calls for a potential mediator. Next, as the literature review has also displayed the promising role of enjoyment in enhancing L2 acquisition, we hypothesized that enjoyment, then, could be the mediator between engagement and flow and flow can both directly and indirectly predict engagement (Figure 1). To test this hypothesis and better understand the interplay between these constructs, we applied a quantum psychology perspective of SLA (Kyriazos and Poga, 2024). Drawing on the quantum entanglement concept, we investigated how flow, enjoyment, and engagement influence each other. To this end, the following research questions are formulated:

1. Does psychological flow have a direct effect on learners’ academic engagement?
2. Does psychological flow have a direct effect on learner enjoyment?
3. Does enjoyment have a direct effect on learners’ academic engagement?
4. Does enjoyment mediate the relationship between psychological flow and academic engagement?
5. What is the total effect of psychological flow on academic engagement, considering both direct and indirect pathways through enjoyment?

Building on Csikszentmihalyi’s (1990) notion that an optimally challenging activity engrosses learners and catalyzes purposeful action, we posit that the deeper a student’s psychological flow, the higher that student’s academic engagement will be. This prediction also aligns with Fredrickson’s (2001) broaden-and-build theory, which holds that positive, absorbing experiences widen attention and fuel subsequent investment in learning tasks. Secondly, when the balance between perceived skill and task challenge is just right—a hallmark of flow—students should experience the foreign-language classroom as intrinsically rewarding and fun. Accordingly, we hypothesize a positive association between psychological flow and foreign-language enjoyment (Csikszentmihalyi, 1990; Dewaele and MacIntyre, 2014). Thirdly, enjoyment is expected to amplify motivation and persistence: positive affect broadens thought–action repertoires and builds enduring personal resources that sustain engagement with academic work (Fredrickson, 2001). We therefore predict that greater foreign-language enjoyment will be linked to higher academic engagement (see also Dewaele and MacIntyre, 2014). Lasly, following the mediation logic articulated by Zhao et al. (2010) and operationalized via bias-corrected bootstrapping procedures recommended by Hayes (2022), we anticipate that psychological flow will influence engagement *solely* through its impact on enjoyment. Once enjoyment is entered into the model, the direct flow → engagement path should diminish to non-significance, indicating full mediation. Together, these hypotheses specify a single indirect pathway—Flow → Enjoyment → Engagement. They are formalized in Figure 1.

## 2 Materials and methods

### 2.1 Research design

This study employed a quantitative, cross-sectional research design to investigate the relationships among psychological flow, enjoyment, and academic engagement in English language classes. This design was chosen over longitudinal or experimental alternatives because it aligned with the objectives of our study. It allowed us to efficiently examine the mediating role of enjoyment in the flow and engagement relationship and to capture their probabilistic interplay at a single point in time using validated scales and path analysis. This design was particularly suitable for mediation analysis when the goal was to test whether a variable, such as enjoyment, mediated the relationship between two others, such as flow and engagement. Moreover, cross-sectional studies though limited in establishing causality offer efficiency in data collection and the ability to examine a probabilistic interplay of constructs simultaneously (Bryman, 2016).

### 2.2 Study context

In Turkish secondary education, English is taught as a compulsory subject, while certain educational policies seek to improve international communication skills and global competitiveness (Öztürk and Aydın, 2019). The study was conducted at a high school in Türkiye where English is taught as the primary foreign language. Classroom activities included social constructivist language learning activities, AI-assisted English speaking and writing classes, often facilitated by AI tools as Call Annie, metaverse, mobile applications and various other digital tools. The physical infrastructure included internet-enabled smartboards and whiteboards. The curriculum followed was National Geographic’s Perspectives series for English language instruction, which focuses on communication skills. All procedures were carried out in accordance with international and institutional ethical guidelines and with the informed consent of the participants’ legal guardians and permissions from school administration was taken.

### 2.3 Participants

A total of 162 Turkish high school students (79 male, 83 female), aged between 14 and 18, were recruited through purposeful sampling, which allowed researchers to select information-rich cases that are relevant to the research questions (Teddlie and Yu, 2007). Practically, it ensured that participants are actively experiencing the constructs under investigation, namely flow in the classroom, enjoyment of language learning, and academic engagement. By targeting students enrolled in English as a compulsory subject, we increased the likelihood that our sample truly represented the population of interest within our educational context.

All participants were enrolled in English as a compulsory foreign language course at the high school level. Proficiency levels were measured to provide a fuller picture of the sample’s language background and to contextualization, which is important in SLA research. Their self-reported language proficiency levels ranged from A2 to C1 on the Common European Framework of Reference for Languages (CEFR). The proficiency levels were collected descriptive purposes only and were not included in the path model. The sample consisted of students from grades 9, 10, 11, and 12. Inclusion criteria required that each participant actively take part in formal English classes at the time of data collection. A power analysis for this study, assuming a medium effect size (0.3), an alpha level of 0.05, and a sample size of 162, revealed a statistical power of approximately 0.77, which was acceptable and indicating a 77% chance of detecting a true effect.

### 2.4 Data collection tools

***Demographic information form:*** this form collected basic participant information, including age, gender, and self-reported proficiency levels. These data helped contextualize participants. No identifying information was gathered.

***Psychological flow scale:*** having developed and validated by Norsworthy et al. (2023), this scale measured students’ psychological flow experiences during learning. It addressed three core dimensions of flow—absorption, effortless control, and intrinsic reward—through three items per dimension, totaling nine items. Each item was rated on a 7-point Likert scale (1 = “Strongly Disagree,” 7 = “Strongly Agree”) (e.g., I was absorbed in the English act/task). The scale thus enabled an objective assessment of how deeply learners immerse themselves in the English learning context, reflecting established constructs of flow within both academic and applied research. Its internal consistency was deemed acceptable (Cronbach’s α = 0.78).

***Academic engagement scale:*** this 15-item scale, developed and validated by Yévenes-Márquez et al. (2022), evaluated students’ engagement to academic tasks from affective, cognitive, and behavioral perspectives. Each item was rated on a 7-point Likert scale (1 = “Strongly Disagree,” 7 = “Strongly Agree”). The instrument comprised three subscales. The instrument consisted of three subscales. Affective Engagement (four items) assessed students’ interest and motivation (e.g., “I am curious about learning new things”). Cognitive Engagement (four items) evaluated the extent to which students actively thought and strove for understanding during learning (e.g., “I try to comprehend as much as possible when reviewing learning activities”). Behavioral Engagement (seven items) captured students’ participation in academic activities and interactions with the teacher (e.g., “I try to answer when the teacher asks questions”). The instrument was used to measure overall engagement, and it had excellent internal consistency (Cronbach’s α = 0.92).

***Foreign language enjoyment scale:*** having developed and validated by Dewaele and MacIntyre (2014), this 21-item instrument measured positive emotional experiences in foreign language learning, including creativity, interest, fun, and pride, as well as the supportive classroom atmosphere arising from teacher-student and peer interactions. Items were rated on a 5-point Likert scale (1 = “Strongly Disagree,” 5 = “Strongly Agree”). The scale included both individual-focused items (e.g., “I can be creative”) and group-oriented items (e.g., “There is a positive atmosphere in class”). It had high internal consistency (Cronbach’s α = 0.86).

While no extensive modifications were made to the scale items themselves (as they have proven reliability and validity), we approached the data analysis with an eye toward non-linear and probabilistic dynamics. The scores derived from these scales were reinterpreted not as static endpoints but as indicators of more fluid underlying processes. Thus, the scale data were not used solely as descriptive purposes but rather to unearth how flow and enjoyment interact to shape engagement, which we grounded in quantum psychology throughout the paper.

### 2.5 Data analysis

We utilized JASP software to analyze data. A path analysis with Maximum Likelihood estimation was performed after screening the data for normality and missing values, confirming that skewness and kurtosis values were acceptable and that internal consistency for all scales exceeded.70. The model examined direct and indirect effects of Psychological Flow on Academic Engagement, with Enjoyment as the hypothesized mediator. In our analysis, we evaluated the goodness-of-fit of our model using several key indices: the Chi-Square (χ^2^) test assessed the overall fit relative to the degrees of freedom; the Comparative Fit Index, with values close to or above 0.95, indicated a good fit; the Root Mean Square Error of Approximation, with values below 0.06, suggested a good model fit; and the Standardized Root Mean Square Residual, with values below 0.08, was deemed acceptable.

Prior to conducting the path analysis, we verified critical assumptions: normality was confirmed as skewness (within ± 1) and kurtosis (within ± 2) values for each variable were acceptable; linearity and homoscedasticity were supported by inspecting scatterplots of residuals, which showed no systematic deviations; multicollinearity was ruled out through examination of Variance Inflation Factors; and measurement error was minimal, as evidenced by high internal consistency (Cronbach’s α > 0.78 for all scales). Additionally, we explored alternative mediation models. In a partial mediation model, we hypothesized that flow influenced engagement both directly and indirectly through enjoyment, but the direct effect was statistically insignificant (β = −0.105, *p* = 0.228), and the model fit indices did not improve with the direct path included. Consequently, the evidence supported a full mediation model, where enjoyment completely mediates the relationship between flow and engagement, a decision driven by both the insignificance of the direct effect and the overall fit of the mediation model.

Although the direct relationship between flow and engagement was non-significant, challenging traditional mediation criteria (Baron and Kenny, 1986; Hair et al., 2014), we adopted the Zhao et al. (2010) framework (and utilized modern bootstrapping techniques as advocated by Hayes (2022) as it emphasizes the significance of the indirect effect and is particularly useful in our case where the independent variable may affect the dependent variable in a fully mediated (or indirect-only) manner, which is consistent with our hypothesis grounded in quantum psychology. We opted against the Sobel test in favor of bootstrapping, given its greater robustness under conditions where distributional assumptions may be violated.

## 3 Results

Skewness and kurtosis values for Flow, Enjoyment, and Engagement were within the acceptable range (± 1 for skewness and ± 2 for kurtosis; Kline, 2016), indicating no substantial deviation from normality. Thus, normality assumptions for path analysis were deemed satisfying. Table 1 presents the descriptive statistics for the key variables.

**TABLE 1:** Descriptive statistics for flow, enjoyment, and engagement.

Variable	*N*	Min	Max	M	SD	Skewness	Kurtosis
Flow	162	2.44	7.00	4.89	0.96	0.541	−0.178
Enjoyment	162	1.42	5.00	4.05	0.75	−0.856	0.628
Engagement	162	1.67	7.00	4.17	1.16	0.206	−0.368

Table 1 showed that students reported moderate to high levels of Flow, Enjoyment, and Engagement. Flow scores ranged from 2.44 to 7.00 (M = 4.89, SD = 0.96) on a 7-point scale, indicating that while most students experienced relatively high flow, some reported lower levels. The slight positive skew (0.54) and near-normal kurtosis (–0.18) suggested a distribution clustered around the mean with a few higher values. Enjoyment scores ranged from 1.42 to 5.00 (M = 4.05, SD = 0.75) on a 5-point scale, reflecting generally high enjoyment among students. The negative skew (−0.86) and moderate positive kurtosis (0.63) indicated that many students reported scores toward the upper end of the scale. Engagement ranged from 1.67 to 7.00 (M = 4.17, SD = 1.16) on a 7-point scale, suggesting a broader range of experiences but overall moderate engagement. The distribution was nearly symmetrical (skew = 0.21) with slightly flattened kurtosis (−0.37), reflecting acceptable normality. Overall, the descriptive statistics in Table 1 support the assumption of normality and justify the use of parametric analyses.

As the data were normally distributed, the path analysis was conducted to examine the relationships among psychological flow (Flow), enjoyment (Enjoyment), and academic engagement (Engagement) in an EFL context. The analysis evaluated both the direct and indirect effects of Flow on Engagement, with Enjoyment serving as a mediator. The direct impact of Flow on Engagement was examined first. Table 2 presents the results.

**TABLE 2:** Direct effects.

					95% confidence interval	
Path	Std. estimate	Std. error	z-value	*P*	Lower	Upper
Flow → Engagement	−0.105	0.087	−1.205	0.228	−0.276	0.066

Estimator is ML.

As shown in Table 2, the direct effect of Flow on Engagement was negative and non-significant (β = −0.105, *p* = 0.228). This result indicated that, when considered in isolation, the level of psychological flow did not exert a statistically significant impact on academic engagement, as the *p*-value was greater than the conventional threshold of 0.05. In practical terms, without including other factors, an increase in flow did not reliably predict changes in engagement. The analysis further examined whether Flow influences Engagement indirectly via Enjoyment. Table 3 presents the indirect effect.

**TABLE 3:** Indirect effects.

					95% confidence interval	
Path	Std. estimate	Std. error	z-value	*P*	Lower	Upper
Flow → Enjoyment → Engagement	0.180	0.049	3.656	< 0.001	0.083	0.276

Estimator is ML.

As shown in Table 3, the indirect effect of Flow on Engagement through Enjoyment was significant (β = 0.180, *p* < 0.001). Psychological flow significantly enhanced enjoyment, which in turn significantly raised academic engagement. The high z-value and the *p*-value well below 0.05 indicated strong evidence for this mediational pathway. Essentially, while flow alone might not drive engagement, it significantly contributed to increasing enjoyment, and this enjoyment was what ultimately boosts engagement in the classroom. To assess the overall relationship between Flow and Engagement, the total effect was examined and is presented in Table 4.

**TABLE 4:** Total effects.

					95% confidence interval	
Path	Std. estimate	Std. error	z-value	*P*	Lower	Upper
Flow → Engagement	0.075	0.078	0.959	0.338	−0.078	0.228

Estimator is ML.

The total effect is a combination of both the direct and indirect effects. The total effect of Flow on Engagement was non-significant (β = 0.075, *p* = 0.338). This may be thought to be an anomaly while the direct pathway (flow → engagement) is statistically non-significant, the indirect path (flow → enjoyment → engagement) is strong and significant. However, it, in fact, indicates that the overall relationship between Flow and Engagement is primarily driven by the indirect effect via Enjoyment rather than a direct influence. In other words, the entire influence of flow on engagement is channeled through the mediator, enjoyment. That is, the positive indirect pathway effectively counterbalances (or outweighs) the minimal and non-significant direct impact, resulting in a scenario of what is described as *full mediation*. The path coefficients for all relationships in the model are summarized in Table 5.

**TABLE 5:** Path coefficients.

					95% confidence interval	
Path	Std. estimate	Std. error	z-value	*P*	Lower	Upper
Enjoyment → Engagement	0.347	0.084	4.153	< 0.001	0.183	0.511
Flow → Engagement	−0.105	0.087	−1.205	0.228	−0.276	0.066
Flow → Enjoyment	0.518	0.057	9.023	< 0.001	0.406	0.631

Estimator is ML.

The results indicate that while the direct effect of Flow on Engagement was non-significant, Flow significantly predicted Enjoyment (β = 0.518, *p* < 0.001). Moreover, Enjoyment had a significant positive effect on Engagement (β = 0.347, *p* < 0.001), suggesting that Enjoyment serves as a mediator in the relationship between Flow and Engagement. Regarding R-squared values (Table 6), approximately 9.4% of the variance in Engagement is accounted for by its predictors, whereas about 26.9% of the variance in Enjoyment is explained by its predictors.

**TABLE 6:** R-squared (R^2^) values for the proportion of variance in the dependent variables explained by the model.

Variable	R^2^
Engagement	0.094
Enjoyment	0.269

**FIGURE 2:**
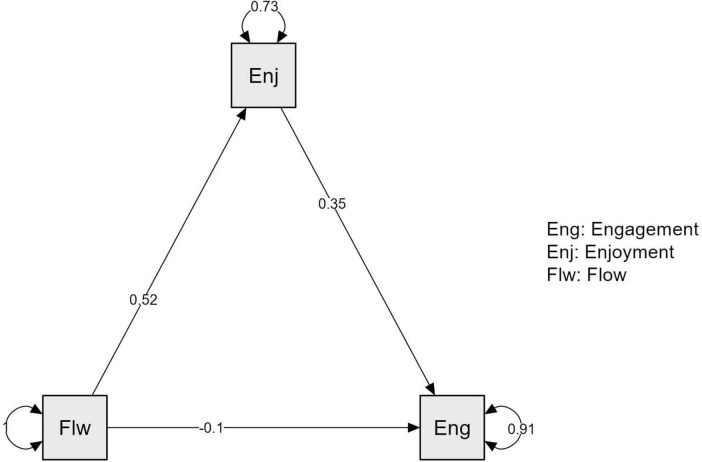
Path plot.

A path plot was formed based on the findings. The relationships among Flow, Enjoyment, and Engagement are visually represented in Figure 2.

The results indicate that psychological flow does not directly influence academic engagement in this EFL context. Instead, Flow significantly enhances Enjoyment, which in turn increases Engagement. The findings suggest that Enjoyment is a key mediator in the relationship between Flow and Engagement.

## Discussion and conclusion

In our study, we shed new light on how psychological flow, enjoyment, and academic engagement relate in L2 learning contexts. Earlier work has often found a direct link between flow and engagement (Jacobs and Morgan, 2022; Li et al., 2021), our findings diverge by showing no significant direct effect. Instead, we found that flow influences engagement indirectly through enjoyment. The observed discrepancy may have stemmed from contextual differences. In our setting, emerging technologies such as AI and the metaverse were frequently integrated into English classes. These tools may have fostered sustained engagement, especially when gamified, playful rewards elicited genuine enjoyment. This could explain our findings, despite the absence of any targeted L2 intervention in the present study. Enjoyment acted as a key mediator, and suggested that engagement stems from affective experiences such as enjoyment rather than purely linear processes, which aligned with other previous research positing that enjoyment fosters cognitive flexibility, persistence, and motivation (Guo, 2021; Hiver et al., 2024).

Our indirect-only result—Flow → Enjoyment → Engagement— is fully compatible with Csikszentmihalyi’s (1990) original eight-component model once we cast that model in probabilistic, quantum-cognition terms. The challenge–skill balance can be read as a superposed cognitive state that collapses into enjoyment when the learner interprets incoming feedback as evidence of mastery. Only after that affective collapse does the behavioral state engagement materialize. Thus, rather than contradicting the flow canon, our data refine it: the autotelic reward is not merely concomitant with flow but functions as the immediate causal driver of engagement in technology-rich L2 classrooms. Our study particularly found a missing puzzle piece in the literature, which strongly and meaningfully added depth to related literature. For instance, in their study with 1044 EFL learners, Dewaele and MacIntyre (2024) suggested that enjoyment was key to creating classroom conditions for flow, yet in their study engagement was not a primary construct under investigation, nor was it directly measured using a separate scale or variable. In another study, Tsang and Dewaele (2023) found enjoyment as a predictor of engagement in EFL context. Strikingly, in our study, we found that flow predicted enjoyment and enjoyment predicted engagement, therefore, flow indirectly predicted engagement with the mediating role of enjoyment with no direct effect of flow on engagement. Through a quantum psychology lens, we gained two fresh perspectives to further interpret the findings and we named them metaphorically with quantum concepts we were inspired by.

First, this finding could either be called as a *wave-function mediation*, in which flow existed as a potential state that collapsed into enjoyment (via emotional resonance), which in turn actualized engagement. We have taken this metaphor from quantum mechanics, where particles exist in a state of probability (a wave function) until they are measured or observed, at which point they collapse into a definite state:

• How we used: Flow is described as a potential or latent state, a condition of immersive, optimal experience that does not directly cause engagement on its own.
• Enjoyment acts as the *measurement* or *collapsing force*—emotional resonance transforms this potential state of flow into a real, conscious emotional experience.
• Result: Once flow collapses into enjoyment, this actualized emotion (enjoyment) becomes the driver of engagement.

Thus, flow sets the stage, but enjoyment is the emotional outcome that mediates flow’s effect on actual academic engagement just as observation collapses a wave function into a real particle. Second, it could be called as *quantum affective entanglement* where emotional states (flow and enjoyment) were entangled, but only one (enjoyment) directly predicted academic engagement. This metaphoric term refers to quantum entanglement, where two particles become linked such that the state of one instantly affects the state of the other, even at a distance.

• How we used: Flow and enjoyment are emotionally entangled, which means they co-exist and influence each other in a complex, possibly inseparable way.
• However, only one of them (enjoyment) is found to directly predict engagement in the data.
• Flow may still be important, but its influence is indirect or latent, working through its entangled relationship with enjoyment.

Therefore, flow and enjoyment are interwoven emotional experiences, but only enjoyment has the observable effect on engagement, which is like how one entangled particle’s state is observable and meaningful, while the other remains hidden.

Our evidence also challenges standard flow theories, which tend to assume a linear pathway from flow to engagement. Instead, we found closer parallels with quantum cognition, which holds that mental states shift probabilistically rather than following fixed outcomes (Feng and Hong, 2022; Pothos and Busemeyer, 2013). Under a quantum psychology lens (Kyriazos and Poga, 2024), enjoyment and flow appeared interwoven, collectively shaping engagement in non-linear ways (Hosseini et al., 2022; Yang et al., 2023).

The findings highlighted the limitations of existing engagement models. Complexity theory often struggled to explain the nature of engagement (Larsen-Freeman, 2023; Zuniga, 2023). Our results pointed to quantum cognition as a promising alternative, recognizing that engagement was best viewed as a probabilistic construct shaped by emotional states, including enjoyment. These insights have clear pedagogical relevance for L2 classes. Since flow alone does not secure engagement, educators should prioritize strategies that promote enjoyment to sustain engagement (Çelik and Baturay, 2024). For instance, gamification, emerging and immersive digital tools can nhance enjoyment and, in turn, engagement (An et al., 2021; Wu et al., 2022). Positive teacher-student and peer relations are also crucial, given their documented influence on both enjoyment and engagement (Hiver et al., 2024; Zheng and Zhou, 2022). Engagement has more to do in L2 classrooms and based on our experience it should be considered as a construct that the teacher has more control over than several others such as anxiety and motivation. By designing lessons in a way that the learners would be interested in and enjoy, they can better support learners’ academic engagement, which would help language learning. In conclusion, we advance our understanding of L2 engagement by confirming that flow’s impact is indirect, occurring through enjoyment. Thus, as in this research, we should move beyond linear interpretations and advocate for a more dynamic, quantum-inspired perspectives in SLA research to better understand the relationships between constructs.

Our findings have indicated that psychological flow does not directly predict academic engagement in L2 learning, contrary to common assumptions in SLA research. Instead, enjoyment emerges as the critical mediator linking flow to engagement, suggesting that L2 learners’ affective states play a decisive role in shaping learning outcomes. These results challenge linear models of learner psychology by indicating that mental states such as flow and enjoyment do not operate in isolation; instead, they interact in a more complex, probabilistic manner. Drawing on the principles of quantum cognition, this study underscores the need for a dynamic, non-deterministic framework that captures the constant interplay of cognitive and emotional factors in SLA. Such a framework expands on the insights of complexity theory, addressing its limitations by highlighting how affective variables like enjoyment can probabilistically influence engagement.

## 5 Limitations

While this study provides valuable insights into Flow, Enjoyment, and Engagement, several limitations should be acknowledged. First, the explained variance in Engagement was relatively low (R^2^ = 0.094), suggesting that other factors may also contribute to engagement. Future research should explore additional mediators and moderators to provide a more comprehensive understanding of engagement in AI-assisted learning environments. The cross-sectional design employed in this study captured a precise snapshot of the probabilistic interplay among flow, enjoyment, and engagement at a single point, though it does not track their dynamic evolution over time, which could be explored in future longitudinal studies. Our study was limited to a specific Turkish educational context, the study can be replicated by further investigating enjoyment as a potential mediator when the results show no direct link between flow and engagement despite expectations. Future studies in other context could help more generalizability of the findings. Our study employed established self-report instruments which is widely accepted in social sciences, but these measures might be somewhat susceptible to biases such as social desirability or recall error, yet the high internal consistency values (α = 0.78–0.92) lend strong support to the reliability of our findings. While our investigation theoretically operationalized quantum psychology with self-reported data, future research must work toward designing and employing time-sensitive tools such as ecological momentary assessments or sensor-based techniques to achieve more robust results.

## 6 Suggestions and implications

The study offers several practical takeaways for language teachers. First, as flow related to task complexity, tasks should be optimally challenging yet also intrinsically rewarding, integrating elements like gamification, personalization, and social interaction to promote *the joy of learning*. Second, the classroom itself is not a controlled experiment: students have complex entanglements of emotions and cognitive states. Learning occurs with less pain in a learning environment, wherein *enjoying the process* becomes spoken. Enjoying learning makes students more inclined to persevere, explore, and deep-engage with the language. Integrating enjoyable, technology-assisted, and flexible activities into lesson plans can create a learning environment for a true engagement where mechanical flow is tempered with a vibrant, dynamic interplay of challenge and pure enjoyment. Third, it also seems true that sometimes engagement is more than just how teachers design an activity, it needs to be a product of positive interpersonal relationships. Thus, teachers are suggested to have a warm teacher-student rapport and support positive peer relationships and classroom community, all of which are factors that can increase enjoyment and overall engagement. Moreover, to enhance academic engagement, educators should prioritize enjoyment alongside flow.

For scholars, the future experimental studies, case studies or practitioner research that offer innovative methods, by employing emerging technologies, to enhance enjoyment can be beneficial. Future studies should also explore longitudinal data to track how enjoyment fluctuates over time and its evolving impact on engagement and how flow acts in this inter between variables when Chaos/Comple action. It is also suggested that a quantum psychology perspective may help to better understand dynamics xity theory falls short. Additionally, it can be explored in future studies how the intersection of Chaos/Complexity and Quantum theory, which may be called as - *Quantum Chaos/Complexity* - in SLA research can help better understand the nature of SLA.

## Data Availability

The raw data supporting the conclusions of this article will be made available by the authors, without undue reservation.
